# Saliency Detection as a Reactive Process: Unexpected Sensory Events Evoke Corticomuscular Coupling

**DOI:** 10.1523/JNEUROSCI.2474-17.2017

**Published:** 2018-02-28

**Authors:** Giacomo Novembre, Vijay M. Pawar, Rory J. Bufacchi, Marina Kilintari, Mandayam Srinivasan, John C. Rothwell, Patrick Haggard, Gian Domenico Iannetti

**Affiliations:** ^1^Department of Neuroscience, Physiology and Pharmacology,; ^2^Department of Computer Science, University College London (United Kingdom),; ^3^Research Laboratory of Electronics, Massachusetts Institute of Technology, Cambridge,; ^4^Institute of Neurology, and; ^5^Institute of Cognitive Neuroscience, University College London (United Kingdom)

**Keywords:** action, EEG, expectancy, force, saliency, sensorimotor integration

## Abstract

Survival in a fast-changing environment requires animals not only to detect unexpected sensory events, but also to react. In humans, these salient sensory events generate large electrocortical responses, which have been traditionally interpreted within the sensory domain. Here we describe a basic physiological mechanism coupling saliency-related cortical responses with motor output. In four experiments conducted on 70 healthy participants, we show that salient substartle sensory stimuli modulate isometric force exertion by human participants, and that this modulation is tightly coupled with electrocortical activity elicited by the same stimuli. We obtained four main results. First, the force modulation follows a complex triphasic pattern consisting of alternating decreases and increases of force, time-locked to stimulus onset. Second, this modulation occurs regardless of the sensory modality of the eliciting stimulus. Third, the magnitude of the force modulation is predicted by the amplitude of the electrocortical activity elicited by the same stimuli. Fourth, both neural and motor effects are not reflexive but depend on contextual factors. Together, these results indicate that sudden environmental stimuli have an immediate effect on motor processing, through a tight corticomuscular coupling. These observations suggest that saliency detection is not merely perceptive but reactive, preparing the animal for subsequent appropriate actions.

**SIGNIFICANCE STATEMENT** Salient events occurring in the environment, regardless of their modalities, elicit large electrical brain responses, dominated by a widespread “vertex” negative-positive potential. This response is the largest synchronization of neural activity that can be recorded from a healthy human being. Current interpretations assume that this vertex potential reflects sensory processes. Contrary to this general assumption, we show that the vertex potential is strongly coupled with a modulation of muscular activity that follows the same pattern. Both the vertex potential and its motor effects are not reflexive but strongly depend on contextual factors. These results reconceptualize the significance of these evoked electrocortical responses, suggesting that saliency detection is not merely perceptive but reactive, preparing the animal for subsequent appropriate actions.

## Introduction

Survival in a fast-changing environment requires animals not only to detect, but also to react, to unexpected events. A large shadow might signal a hawk, or a rustling in the bush might signal a nearby prey. In a split second, an animal must identify the salient stimulus and react with the appropriate behavioral response.

To initiate these behavioral responses rapidly, an animal must build expectations about the structure of its sensory environment, and thereby detect changes violating these expectations, both at low level (e.g., detection of stimulus edges through lateral inhibition) (Blakemore et al., 1970) and high level (e.g., detection of deviant stimuli embedded within a stream of standard stimuli) (Garrido et al., 2013). Influential theories of brain function suggest that dedicated brain networks construct and continuously update predictive models of the environment (Schultz et al., 1997; Engel et al., 2001; Friston, 2010). By comparing these models with the actual sensory input, animals can detect environmental changes, also known by the related terms violation, mismatch, surprise, or saliency, to update predictions, trigger actions, or both (Näätänen et al., 2007; Morrison et al., 2013; Ullsperger et al., 2014; Wessel and Aron, 2017). Both computational aspects and physiological implementation of predictive models have been described in a variety of animal systems (Rao and Ballard, 1999; Ulanovsky et al., 2003; Yaron et al., 2012; Garrido et al., 2013).

In humans, it is well known that salient and fast-rising sensory events generate a remarkably large neural synchronization, which manifests itself as a biphasic EEG potential, widespread and maximum over the scalp vertex (Walter, 1964; Mouraux and Iannetti, 2009). This biphasic vertex wave, which is evoked by stimuli of any modality provided that they are salient enough (Bancaud et al., 1953; Mouraux and Iannetti, 2009), has largely been interpreted as a byproduct of saliency detection. However, it is unknown whether the vertex wave also impacts the motor control system to prepare a potential behavioral response. This lack of knowledge is surprising given that survival ultimately depends not only upon detecting unexpected events, but also on initiating appropriate behavioral responses.

Here we report a direct link between salient stimuli, brain activity, and behavior in healthy humans. We take advantage of a novel, highly sensitive transduction device to record fine-scale variations of isometric force exerted by the fingers, with millisecond precision. We delivered sudden (i.e., fast-rising) and temporally unexpected stimuli of different sensory modalities while measuring EEG and EMG activity. We found that mild, substartle but still salient stimuli modulated the applied isometric force in a multiphasic pattern predicted by the EEG signals. The force modulation and EEG signals did not, however, simply reflect peripheral afferent input, but depended on stimulus saliency. That the same EEG response reflects sensory processing while unavoidably triggering a motor response, suggests that sensory and motor processing are intimately entwined and that saliency detection should be reinterpreted as a reactive process rather than a perceptive one.

## Materials and Methods

### 

#### 

##### Subjects.

A total of 70 healthy human participants (34 males, mean ± SD age, 22.9 ± 3.3 years, age range 18–41 years) took part in four experiments (*N* = 18, 28, 14, and 10, respectively). All participants gave written informed consent and were paid for their participation. All procedures were approved by the ethics committee of University College London.

##### Sensory stimulation.

Auditory stimuli consisted of a fast-rising tone (rise and fall time 5 ms, frequency 4000 Hz, duration 50 ms), which was presented through a single CAT LEB-401 loudspeaker. The loudspeaker was placed in front of the left hand of the participant. Electrical stimuli (duration 200 μs) were delivered transcutaneously to the left median nerve at the wrist, with a constant-current stimulator (Digitimer DS7A) controlled using a National Instrument card (USB-6008).

The intensity of both auditory and electrical stimulations was adjusted individually before the beginning of each experiment and is below referred to as low, middle, and high. High-intensity stimulations (used in Experiments 2–4) corresponded to the highest loudness (auditory stimulations) or current (electrical stimulations) that the participants could tolerate without feeling discomfort or pain, and that did not evoke an EMG response in the sternocleidomastoid muscle. Middle- and low-stimulus intensities (used in Experiments 1 and 2) were 60% and 20% of the high-stimulus intensity, respectively. Finally, the intensity of the startling auditory stimuli delivered in Experiment 4 was 100 dB (in comparison, the intensity of the high but not startling auditory stimuli used in Experiment 3 never exceeded 70 dB).

Stimuli were delivered using Presentation (Neurobehavioral Systems). Triggers synchronized with stimulus onset were sent to two computers used for acquiring force and EEG data.

##### Force recording.

**Figure 1.**
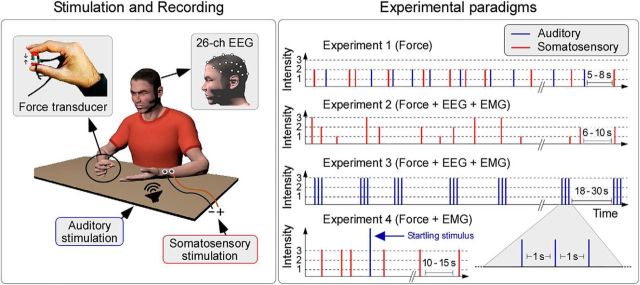
Experimental methods and paradigms. Left, Participants were instructed to perform an isometric motor task: applying a constant force on a transducer using the thumb and index finger of the right hand, while keeping their eyes closed. Meanwhile, we delivered either somatosensory stimuli (via electrical stimulation of the left median nerve) or acoustic stimuli (through a loudspeaker placed close to the participant's left hand). Right, In Experiment 1, we delivered somatosensory and acoustic stimuli (medium intensity). In Experiment 2, we delivered somatosensory stimuli (low, medium and high intensity). In Experiment 3, we delivered auditory stimuli (high intensity). In Experiment 4, we delivered standard somatosensory stimuli (high intensity) and startling auditory stimuli (100 dB). Force was recorded in all experiments. EEG was recorded in Experiments 2 and 3. EMG was recorded in Experiments 2–4.

The force applied by the participants (see Experimental design) was sampled using a force-torque (F/T) transducer (ATI nano17, Industrial Automation). This device measures mechanical responses using silicon strain gauges within a monolithic design to provide high stiffness characteristics while protecting against noise. The device allows recording six components of force and torque (Fx, Fy, Fz, Tx, Ty, Tz). The Fz component represented the direction toward which participants were instructed to exert the force while holding the transducer (see Fig. 1), and it was the source of the data reported hereafter. The transducer was connected to a data acquisition card (National Instruments 6363) through which the sensor data from the silicon strain gauges was converted into F/T information based upon calibrated values established by the manufacturer. At the start of each recording session, the F/T information was set to zero to mitigate the effects of potential sensor drifts. Data were sampled at 500 Hz with unique timestamps to allow synchronization with the stimulation triggers. To facilitate two-finger grip, the transducer was mounted in between two plastic cylindrical extensions (see Fig. 1).

##### EEG recording.

The EEG was recorded using a 32-channel amplifier (SD-32, Micromed) at a sampling rate of 1024 Hz. The EEG was recorded from 26 Ag-AgCl electrodes placed on the scalp according to the International 10–20 system and referenced to the nose. Electrode positions were Fp1, Fpz, Fp2, F7, F3, Fz, F4, F8, T3, C3, Cz, C4, T4, T5, P3, Pz, P4, T6, O1, Oz, O2, FCz, FC4, FC3, Cp3, and Cp4 (Sharbrough et al., 1991). The electro-oculogram was recorded from two pairs of surface Ag-AgCl electrodes, each placed laterally to the outer canthus. Impedances were kept <10 kΩ.

##### EMG recording.

The remaining channels of the EEG amplifier were dedicated to recording the EMG, using four pairs of surface Ag-AgCl electrodes, using a bipolar montage. In Experiments 2 and 3, we recorded the EMG from the right sternocleidomastoid, biceps, triceps, and first dorsal interosseous muscles. In Experiment 4, we recorded the EMG from the right and left sternocleidomastoid muscles.

##### Experimental design.

In all experiments, participants were sitting in front of a table, with the ulnar aspect of the forearm and of the hand resting on the table surface. They were asked to exert a constant isometric force on the transducer, which was held between the index finger and thumb of the right hand, as illustrated in Figure 1.

All experiments consisted of several blocks. Before each block, participants were instructed to keep their eyes closed (to minimize distraction and reduce eye movements) and exert a gradually increasing force, until they reached a level between 1 and 2 N. At the beginning of each block, feedback to the participants was provided verbally by the experimenters, who could read the measured force in real time: once the correct level was reached, participants were instructed to keep the force applied as constant as possible throughout the recording blocks, and keep their eyes closed. During each block, participants received either auditory or electrical stimuli, as detailed below. There was a short pause of ∼5–10 s between consecutive blocks. A schematic representation of the stimuli delivered in each experiment is given in Figure 1.

In Experiment 1 (18 participants), auditory and electrical stimuli, all of middle intensity, were delivered. Each block comprised between 4 and 6 stimuli presented in randomized order with an interstimulus interval of 5–8 s (rectangular distribution). The total number of blocks was 8. In total, participants received 40 stimuli, 20 per modality.

In Experiment 2 (28 participants), only electrical stimuli, but of three energies, were delivered. Each block comprised 5–7 stimuli presented in randomized order with an interstimulus interval of 6–10 s (rectangular distribution). The total number of blocks was 7. In total, participants received 42 stimuli, 14 for each intensity.

In Experiment 3 (14 participants), only auditory stimuli, all of high intensity, were delivered. Stimuli were delivered in trains of 3 (S1, S2, and S3: a triplet) at a constant interval of 1 s (i.e., at 1 Hz). The time interval between each triplet ranged between 18 and 30 s (rectangular distribution). The total number of trials was 30, for a total of 90 stimuli.

Finally, in Experiment 4 (10 participants), we delivered 28 standard electrical stimuli (all of high intensity) and 4 exceptionally loud (100 dB) startling auditory stimuli. Each block comprised 4 stimuli. There was never more than one startling stimulus per block, and there were never more than two consecutive blocks containing a startling stimulus. Hence, the startling stimuli had longer intertrial intervals, higher intensity, and were presented less frequently than the other standard stimuli used in the previous experiments (Brown et al., 1991; Yeomans and Frankland, 1995; Fernandez-Del-Olmo et al., 2013). The interstimulus interval ranged between 10 and 15 s (rectangular distribution). The number of blocks was 8, which resulted in a total of 32 stimuli presented across the experiment.

##### Force data processing.

In all experiments, force magnitude time series were first interpolated to obtain a regular sampling rate of 1000 Hz. Continuous data were segmented into epochs of 3.4 s (−0.4 to 3 s relative to stimulus onset). Each epoch was detrended using the prestimulus interval (Tracy, 2007; Welsh et al., 2007). In Experiments 1–3, trials contaminated by artifacts (±0.3N from the mean of the prestimulus interval) or deviating >3 SDs from the participant's mean exerted force across all trials were excluded from further analyses. The corresponding EEG and EMG trials were also excluded. These trials constituted 10.03% of the total number of trials. Finally, epochs were baseline corrected using the −0.05 to 0 s prestimulus interval.

**Figure 2.**
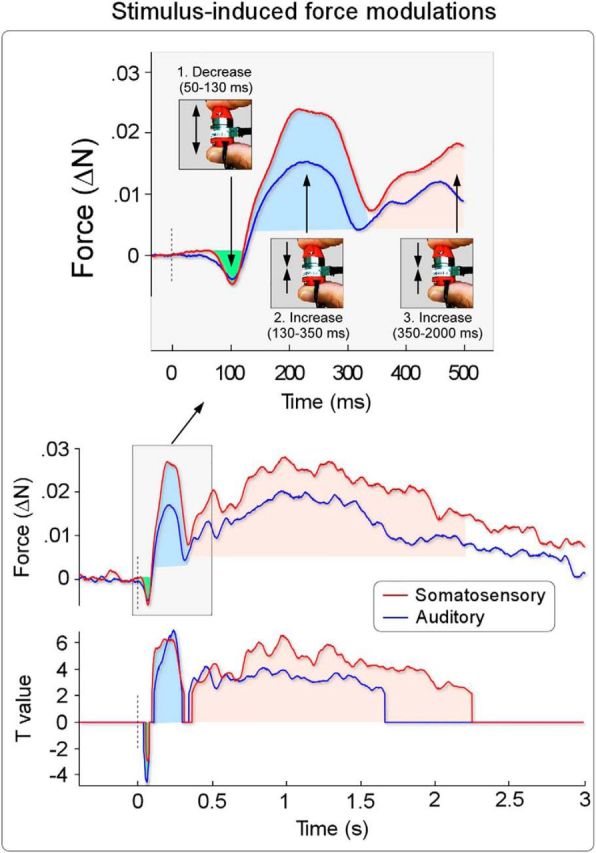
Stimulus-induced modulation of force magnitude. Experiment 1 (*n* = 18). Somatosensory and auditory stimuli were delivered while participants were exerting a constant force of ∼1 N. Middle and Top, Both somatosensory (red) and auditory stimuli (blue) elicited a consistent modulation of the applied force in a complex triphasic pattern. Stimuli first elicited a decrease in force (green area), peaking at ∼100 ms, immediately followed by a longer-lasting increase (blue area) peaking at ∼250 ms. The two initial modulations (enlarged in the top) were followed by a third increase in force (pink area), starting at ∼350 ms after stimulus and lasting until ∼2 s after stimulus. Bottom, Time course of *t* values showing the intervals in which the modulation was consistent across participants (one-sample *t* test against baseline, threshold *p* = 0.05).

Given that, in Experiments 1 and 2, a slow-rising stimulus-evoked force modulation lasting up to 2.5 s was observed (see Fig. 2), in Experiment 3 (i.e., the only experiment in which stimuli were repeated at 1 Hz), epochs were bandpass filtered at 1–30 Hz. This allowed a robust estimation of the magnitude of the transient responses of force magnitude elicited by each stimulus composing the triplet.

##### EEG processing.

Continuous EEG data were first bandpass filtered at 0.5–30 Hz (Butterworth, third order), and then segmented into epochs of 3.4 s (−0.4 to 3 s relative to stimulus onset). Artifacts due to eye blinks or eye movements were subtracted using a validated method based on an independent component analysis (Jung et al., 2000). In all datasets, independent components related to eye movements had a large electro-oculogram channel contribution and a frontal scalp distribution. To match the sampling rate of the force time-series, EEG epochs were downsampled to 1000 Hz. Finally, epochs were baseline corrected using the −0.4 to 0 s prestimulus interval.

##### EMG processing.

Continuous EMG data were first high-pass filtered at 55 Hz (Butterworth, third order), and then segmented into epochs of 3.4 s (−0.4 to 3 s relative to stimulus onset). Epochs were downsampled to 1000 Hz, full-wave rectified, and baseline corrected using the −0.4 to 0 s prestimulus interval.

##### Statistical analysis.

In Experiment 1 (force only), epochs belonging to the same experimental condition (i.e., somatosensory or auditory) were averaged together, thus yielding two average waveforms per participant. To assess the consistency of stimulus-induced modulation of force magnitude over time, a one-sample *t* test against zero (i.e., against baseline) was performed for each time point of the entire waveform. This analysis yielded, for each modality, a time-series of *t* values.

In Experiment 2 (force, EEG, EMG), the presence of a relationship between the variability of the EEG and force signals was first assessed within participant (i.e., on a trial-by-trial level). To achieve this, each EEG and force trial was smoothed using a sliding window of 20 ms, moving in steps of 10 ms. Next, the trial-by-trial correlation coefficient (Pearson's *r*) was computed between EEG amplitude and force magnitude, for all possible pairs of time points between the interval −50 to 400 ms of the EEG time course (i.e., the interval encompassing both the vertex negativity and positivity) and the interval −50 to 2000 ms of the force time course (i.e., the interval encompassing both the force decrease and the two following force increases). Possible effects due to changes of afferent input were partialled out by adding stimulus intensity as a control variable. This resulted in 26 correlation matrixes (one for each EEG electrode), each consisting of 45 × 205 values. This analysis allowed identifying possible signal changes in one measure (either EEG amplitude or force magnitude) that correlated with simultaneous, later, or earlier changes in the other measure.

To assess the consistency of such trial-by-trial correlations across participants, the coefficients (one Fisher's z-transformed Pearson's *r* for each participant) were contrasted against zero using one-sample *t* tests, one for each pixel of the matrix. Cluster-based permutation testing (Maris and Oostenveld, 2007) was used to account for multiple comparisons across time points and EEG electrodes. Therefore, clusters were based on both temporal consecutivity and spatial adjacency of EEG electrodes. A cluster had to be composed of at least two consecutive time points with a *p* value <0.05 on at least three neighboring EEG electrodes. The significance value of each cluster corresponded to the sum of all *t* values of the pixels composing it. Once these clusters were identified, permutation testing was used to assess their significance. Specifically, 1000 permutations of the data were used to generate a random distribution of cluster significances. This random distribution was used to define a threshold (*p* = 0.05) against which the actual significant clusters were assessed. Thus, only the pixels surviving both thresholds (consecutivity in time and adjacency in space, as well as random permutations) were considered significant.

The relationship between the variability of EEG and force signals was also explored between participants. Thus, we tested whether participants showing overall larger EEG responses also showed larger force responses. The same analysis strategy used to explore the within-participants EEG-force correlations was applied. First, single trials within each participant were averaged, thus yielding 26 pairs of waveforms for each participant (1 pair for each EEG electrode). Next, for each pair, the correlation between all possible pairs of time points was computed. This resulted in 26 correlation matrixes (one for each electrode), each consisting of 45 × 205 elements.

Matrix elements representing a significant correlation (*p* < 0.05) in at least two consecutive time points and on at least three neighboring EEG electrodes formed a significant cluster. The significance value of each cluster corresponded to the sum of all Pearson's *r* values of the pixels composing it. Once these clusters were identified, permutation testing was used to assess their significance, as described above.

EMG epochs of each participant were averaged across trials, thus yielding one average waveform for each muscle and participant. To assess the across-subject consistency of possible stimulus-induced modulation of EMG over time, a one-sample *t* test against zero (i.e., against the average of the baseline) was performed for each time point of the entire waveform.

In Experiment 3 (force, EEG, EMG), EEG epochs were averaged across trials, time-locked to the onset of the first stimulus of the triplet (S1). In each participant, the amplitude of the auditory-evoked negative (N) and positive (P) peaks of the vertex wave at Cz was measured, for each stimulus of the triplet. N and P waves were defined as the most negative and positive deflections following the onset of each stimulus.

Force epochs were also averaged across trials, time-locked to the onset of the first stimulus of the triplet (S1). In each participant, the peak magnitude of the auditory-evoked force decrease was measured, as well as the following force increase elicited by each stimulus of the triplet.

To assess the modulation of both EEG and force induced by stimulus repetition, two one-way ANOVAs were performed, one for EEG and one for force, with the experimental factor “stimulus repetition” (three levels: S1, S2, S3). When the main effect was significant, pairs of conditions were compared using paired-sample *t* tests.

EMG epochs were analyzed with the same procedure described for Experiment 2. In addition, a one-way ANOVA was performed, with the experimental factor “stimulus repetition” (three levels: S1, S2, S3).

In Experiment 4 (force, EMG), both force and EMG epochs belonging to the same experimental condition (i.e., standard or startling) were averaged across trials. To assess the consistency of the stimulus-induced modulation of force magnitude and EMG activity over time, a one-sample *t* test against zero (i.e., against baseline) was performed for each time point of the waveform.

## Results

### Salient stimuli modulate voluntarily exerted force

To determine whether sudden somatosensory and auditory stimuli can modulate voluntarily exerted forces, we delivered fast-rising stimuli of two different modalities (somatosensory and auditory) while participants were asked to exert a constant isometric force on a transducer held in their hand (Fig. 1, Experiment 1). We used stimuli of mild intensity to prevent overt, startle-like motor responses. Force was measured using a transducer with millinewton resolution.

We observed that, regardless of their modality, the stimuli elicited a consistent force modulation in a complex triphasic pattern composed of an initial force reduction followed by two distinct force increases (Fig. 2). In particular, the stimulus first elicited a small reduction of the applied force (−7 ± 7 mN [somatosensory stimulus]; −6 ± 5 mN [auditory stimulus]) peaking at ∼100 ms after stimulus (range: 50–130 ms). This first decrease was immediately followed by a larger, longer-lasting increase of force (33 ± 24 mN [somatosensory]; 22 ± 13 mN [auditory]), peaking at ∼250 ms after stimulus (range: 130–350 ms). These two initial modulations were followed by a third, much longer-lasting increase of the applied force (23 ± 18 mN [somatosensory]; 15 ± 20 mN [auditory]), starting at ∼350 ms after stimulus and lasting until ∼2 s after stimulus. This third modulation was sustained and therefore did not have a clearly identifiable peak.

Given that participants exerted a baseline force of ∼1 N, the magnitude of the three modulations was ∼−0.7%, +3.3%, and +2.3% (somatosensory) and −0.6%, +2.2%, and +1.5% (auditory) of the baseline.

**Figure 3.**
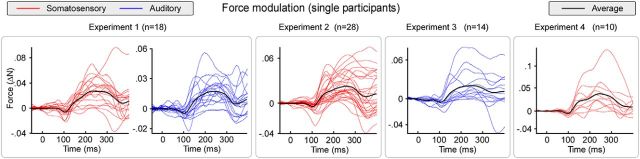
Time courses of force modulation in individual participants. Force modulation by transient somatosensory (red) or auditory (blue) stimuli, in 70 human participants. Left to right, Plots represent the data from Experiments 1–4. In all experiments, and nearly in all participants, both somatosensory and auditory stimuli elicited a consistent modulation of the exerted force. This modulation consisted of a complex triphasic pattern. A first force decrease (∼100 ms) was immediately followed by a longer-lasting force increase (∼250 ms). These first two modulations were followed by a third force increase (∼350 ms), lasting until ∼2 s after the stimulus (data not shown).

Point-by-point one-sample *t* tests confirmed that these three force modulations were consistent across the 18 participants of this experiment (Fig. 2). Single-subject waveforms showing the force modulation are displayed in Figure 3.

These results indicate that sudden environmental changes have an immediate effect on motor reactivity, as reflected in the evoked modulation of applied force's magnitude. The complex and multiphasic nature of the observed force-modulation pattern suggests that salient sensory events trigger a reactive, rather than a perceptive, process.

### Force modulation is coupled to cortical activity

**Figure 4.**
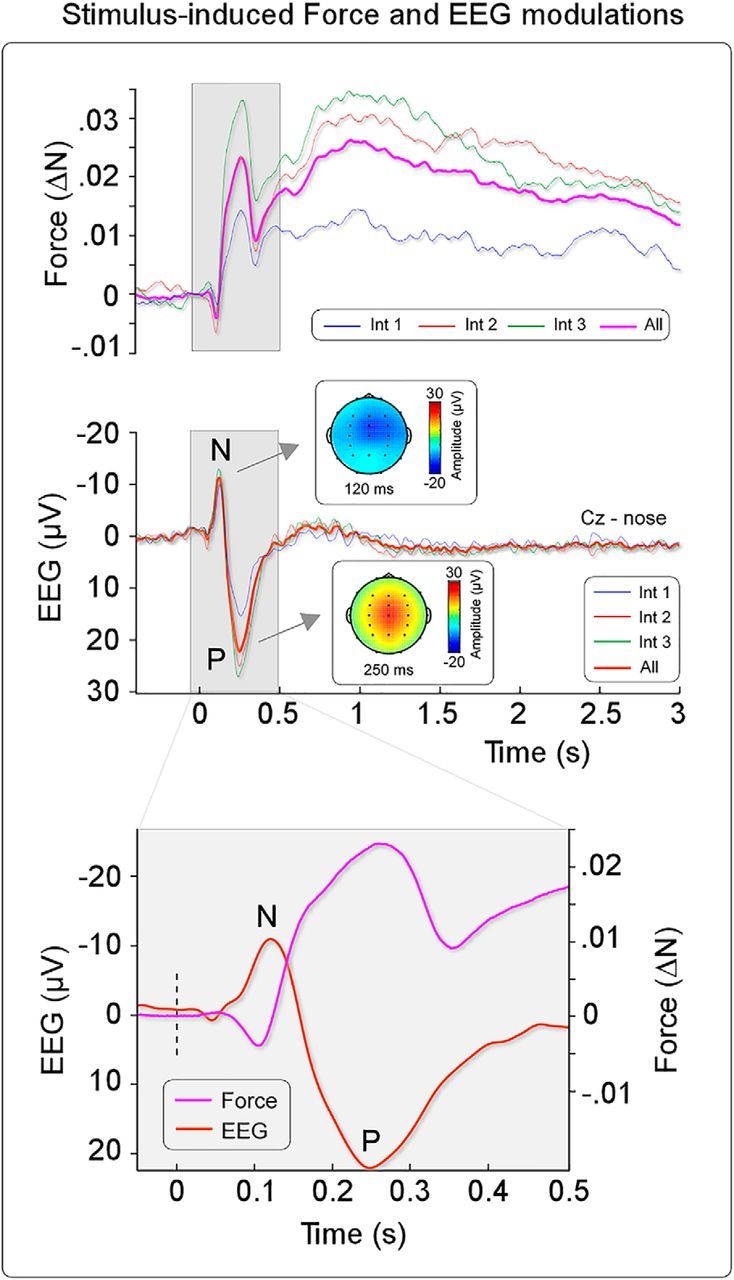
Stimulus-induced force and EEG modulations. Top, Modulations of the applied force elicited by graded somatosensory stimulation. Experiment 2 confirmed in a different group of 28 participants the triphasic force modulation observed in Experiment 1 (Fig. 2). Latency, magnitude, and durations of the three components were similar in the two experiments. Middle, The same graded somatosensory stimuli also elicited the typical biphasic negative (N) and positive (P) waves, maximal at the scalp vertex, peaking at ∼120 and ∼250 ms after stimulus, respectively (displayed signals were recorded at Cz). Scalp distributions are shown in the insets. Bottom, EEG (orange) and force (purple) signals superimposed in the −50 to 500 ms time window. Both signals are composed of two consecutive quasi-simultaneous peaks of opposite polarity.

Previous studies have shown that salient stimuli evoke well-described potentials in the human EEG (Treede et al., 1988; Liang et al., 2010). Yet, how these responses might regulate motor reactivity is unknown. To investigate this relationship, we assessed whether the force modulation observed in the task above was coupled with cortical activity. We administered our force-modulation task (triggered by somatosensory stimuli) while measuring brain activity with EEG (Fig. 1, Experiment 2). To ensure the reproducibility of Experiment 1's findings, we conducted this experiment in a different cohort of 28 participants. We confirmed that somatosensory stimuli elicited the triphasic modulation of the force applied on the transducer (Fig. 4). Latency, magnitude, and durations of all three components were similar to those observed in the previous cohort (Figs. 2–4). In this experiment, we also measured EMG activity to rule out startle-like responses (see also Experiment 4 below and Fig. 7).

Somatosensory stimuli elicited large deflections in the EEG (Fig. 4, middle and bottom). The largest response was the typical biphasic negative-positive wave, maximal at the scalp vertex: the negative wave peaked at ∼120 ms after stimulus, and the positive wave peaked at ∼250 ms after stimulus. These results are consistent with previous reports of vertex potentials elicited by transient somatosensory stimuli (Walter, 1964; Mouraux and Iannetti, 2009; Liang et al., 2010). Visual inspection of the time course of these two signals suggests that cortical activity is coupled with the motor response: the peak latencies of the first two force modulations approximately corresponded with those of the negative and positive vertex waves (Fig. 4, bottom). Nevertheless, caution is needed in interpreting evoked potential latencies because cortical generators of scalp potential could act as leaky integrators, blurring the exact timing of sensory processing (Eliasmith and Anderson, 2004; Graben et al., 2007).

These results, however, suggest that cortical activity could drive the motor response. If so, we would expect that (1) within an individual, trial-by-trial cortical responses would correlate with trial-by-trial force modulations; and (2) across the population, large cortical responses would be predictive of strong force modulations.

#### Within-participant EEG-force correlations

To determine whether individual cortical responses varied with force modulations, we examined correlations between EEG and force signals across all time points. We found strong evidence that trial-to-trial variability of the EEG signal at multiple time points in the vertex wave matched the trial-to-trial force modulation.

**Figure 5.**
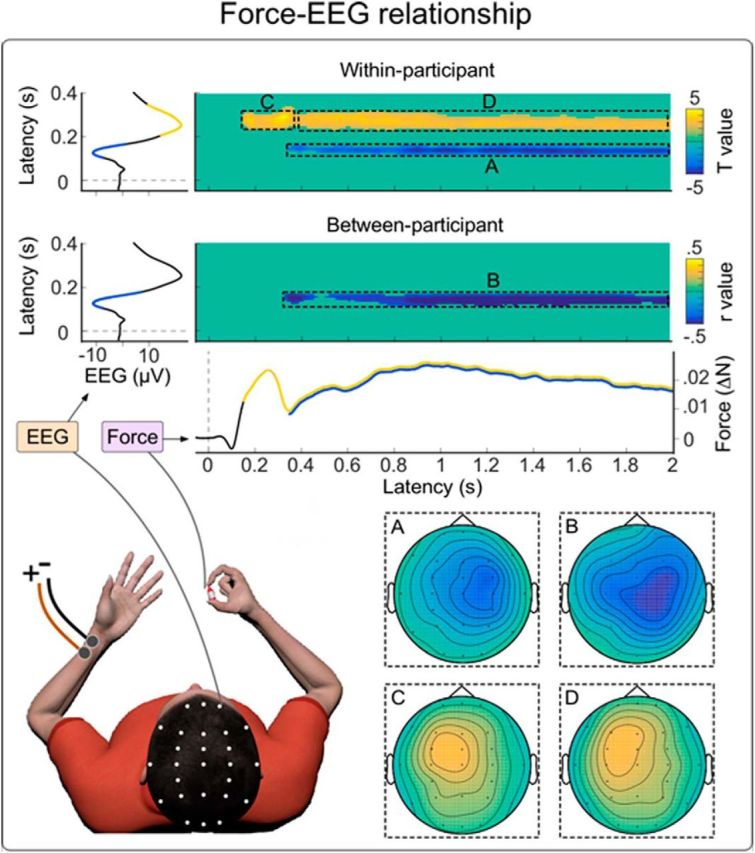
Within- and between-participants relationship between EEG and force signals. Top, Bidimensional plot represents the *t* values reflecting the significant trial-by-trial correlation (Pearson's *r*) between EEG and force, for all possible pairs of time points, at electrode Cz. Significant pixels survived a threshold for both temporal consecutivity and spatial adjacency across scalp electrodes (cluster-based permutation testing). This analysis allowed identifying signal changes in one measure (either EEG amplitude or force magnitude) that predict or are predicted by simultaneous, later, or earlier changes in the other measure. EEG and force time courses are shown on the *y*-axis and the *x*-axis, respectively. There was a tight relationship between trial-by-trial variability of EEG and force. The EEG time interval 110–180 ms, corresponding to the vertex wave negativity, was negatively correlated with the force variability in the time window ∼300–2000 ms, corresponding to the late force increase. The topography of this cluster (inset ***A***) was lateralized toward the hemisphere contralateral to the stimulated hand (i.e., the hemisphere ipsilateral to the hand exerting the force). The EEG time interval ∼200–370 ms, corresponding to the vertex wave positivity, was positively correlated with the variability in force magnitude in the time window ∼130–2000 ms, which was the period encompassing both the early and the late increases. During the first (***C***) and second (***D***) increases, this relationship was strongest at central electrodes, particularly over the hemisphere contralateral to the hand exerting the force (i.e., the hemisphere ipsilateral to the applied stimulus) (insets ***C***, ***D***). Bottom, Bidimensional plot represents the Pearson's *r* values reflecting the significant between-participant correlation between EEG and force, at electrode Cz. The EEG time interval ∼110–180 ms, corresponding to the vertex wave negativity, was negatively correlated with the variability in force magnitude in the time window ∼300–2000 ms, corresponding to the late force increase. The topography of this cluster (inset ***B***) was lateralized toward the hemisphere contralateral to the stimulated hand (i.e., the hemisphere ipsilateral to the hand exerting the force).

During the vertex wave negativity (∼110–180 ms), EEG variability was negatively correlated with the force magnitude in the time window ∼300–2000 ms (cluster *p* = 0.019, cluster-corrected; *r* value mean: −0.10; *r* value range: −0.18 to −0.05). This time period encompasses the late force increase (Fig. 5*A*). This correlation between the EEG negativity and the force increase was strongest over the central scalp electrodes, particularly in the hemisphere contralateral to the applied stimulus (i.e., ipsilateral to the hand exerting the force). This lateralization was confirmed by a *t* test comparing the Pearson's *r* values averaged across the right central electrodes (C4, Cp4, and FC4) with those averaged across the left central electrodes (C3, Cp3, and FC3) (*t*_(27)_ = 2.8, *p* = 0.022; Fig. 5*A*).

Similarly, during the vertex wave positivity (∼200–370 ms) EEG variability was positively correlated with force magnitude in the time window ∼130–2000 ms, corresponding to both the early and the late force increases (cluster *p* = 0.0009, cluster-corrected; *r* value mean: 0.1; *r* value range: 0.06–0.19) (Fig. 5*C*,*D*). The scalp distribution of this correlation was maximal over the central electrodes ipsilateral to the applied stimulus (i.e., contralateral to the hand exerting the force). This lateralization was confirmed by a *t* test comparing the Pearson's *r* values averaged across the right central electrodes (C4, Cp4, and FC4) with those averaged across the left central electrodes (C3, Cp3, and FC3) (*t*_(27)_ = 2.8, *p* = 0.009; Fig. 5*C*,*D*).

#### Between-participants EEG-force correlations

If cortical activity drives the motor response, we would also expect that participants displaying relatively larger EEG waves would show larger force modulations. We observed that the across-subject EEG variability in the time interval ∼110–180 ms, corresponding to the vertex wave negativity, was negatively correlated with the variability in force magnitude in the time window ∼300–2000 ms, corresponding to the late increase of force magnitude (cluster *p*=0.043, cluster-corrected) (Fig. 5*B*). This cluster was analogous to the one reflecting the negative correlation between EEG and force trial-by-trial variability observed in the within-participants analysis. Likewise, the scalp distribution of this correlation was maximal over the central electrodes in the hemisphere contralateral to the applied stimulus (i.e., ipsilateral to the hand exerting the force). The presence of both within- and between-participants correlations is remarkable. Indeed, between-subject correlations are more rarely observed than within-subject correlations (e.g., Iannetti et al., 2005; Hanslmayr et al., 2005, 2007), and collectively suggest a stronger relationship between the physiological measure and the behavioral effect.

Together, these data show that the magnitude of force modulation strongly correlates with the amplitude of the cortical activity elicited by the same stimuli, both within-participant (trial-by-trial) and between-participants. These results suggest that it is the cortical activity underpinning the vertex wave that drives the force modulation.

### Force and cortical modulations reflect stimulus saliency rather than afferent input

To determine whether the cortical and force modulations depend upon the stimulus context or the afferent sensory input, we used a validated paradigm that dissociates stimulus saliency from the intensity of the afferent volley (Iannetti et al., 2008; Valentini et al., 2011). This paradigm consists of the repetition of three identical auditory stimuli at 1 Hz (a triplet: S1-S2-S3), where S1 is more salient than S2 and S3. Importantly, all stimuli are physically equal (Fig. 1, Experiment 3). If the observed force and cortical modulations simply reflect the peripheral afferent volley, we would expect the same magnitude in the responses elicited by S1, S2, and S3. If the force and cortical modulation instead reflect stimulus saliency, we would expect both modulations to be enhanced in response to S1 compared with S2 and S3.

**Figure 6.**
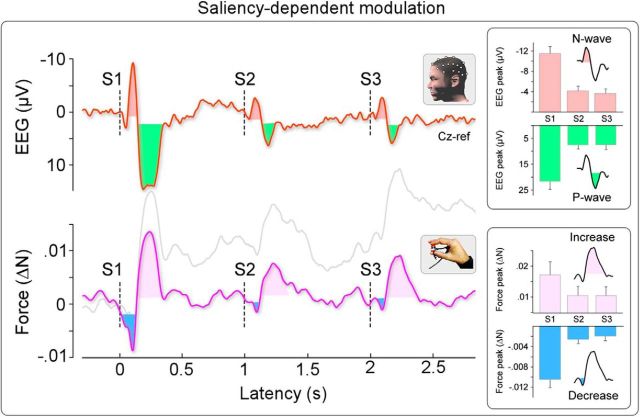
Stimulus-evoked EEG and force responses depend on context. Experiment 3 (*n* = 14). EEG amplitude at Cz (top) and force magnitude (bottom) during the repetition of three identical auditory stimuli (S1-S2-S3) at 1 Hz. Top, Stimulus repetition of three auditory stimuli robustly modulated the amplitude of both the negativity and the positivity of the vertex wave, which were significantly smaller in the response elicited by S2 and S3 compared with S1. Bottom, The same auditory stimuli also modulated the magnitude of the early force decrease and increase. Both force responses were significantly reduced in the response elicited by S2 and S3. Force data were bandpass filtered at 1–30 Hz to avoid the influence of the third long-lasting modulation elicited by S1 on the two early short-lasting modulations elicited by S2 and S3. Pale gray line shows non–bandpass-filtered time course of exerted force, highlighting the consistency of force modulations across all experiments. Thus, Experiment 3 not only further confirmed in a different group of 14 participants the force modulation observed in Experiments 1 and 2 (see Figs. 2, 4) but also provided compelling evidence that both the EEG and the force signals are similarly affected by contextual factors, and both depend on the stimulus behavioral relevance. Error bars indicate 1 standard error of the mean.

As expected, the magnitude of both the negativity (N) and the positivity (P) of the vertex wave was significantly reduced in the response elicited by S2 and S3 compared with the magnitude of the responses elicited by S1 (Fig. 6, top right). One-way ANOVA showed strong evidence for an effect of “stimulus repetition” (N: *F*_(2,26)_ = 44.5, *p* < 0.001; P: *F*_(2,26)_ = 54.8, *p* < 0.001). *Post hoc t* tests revealed that the amplitude of the responses elicited by S2 and S3 was significantly reduced compared with the amplitude of the response elicited by S1 (N: *p* < 0.001; P: *p* < 0.001, for both S1 vs S2 and S1 vs S3).

Remarkably, the force response mirrored the neural response. We found that the repetition of three auditory stimuli at 1 Hz also modulated the magnitude of the early force decrease and increase. Both modulations were significantly reduced in the response elicited by S2 and S3 (Fig. 6, bottom right). One-way ANOVA showed strong evidence for an effect of “stimulus repetition” (force decrease: *F*_(2,26)_ = 32.89, *p* < 0.001; force increase: *F*_(2,26)_ = 8.59, *p* < 0.01). *Post hoc t* tests revealed that the magnitude of the responses elicited by S2 and S3 was significantly reduced compared with the magnitude of the corresponding responses elicited by S1 (force decrease: *p* < 0.001; force increase: *p* < 0.012, for both S1 vs S2 and S1 vs S3).

These results provide compelling evidence that both the force and the cortical modulations are related to stimulus saliency rather than peripheral afferent input. Thus, it is clear that both the cortical and muscular responses are not stereotyped but strongly depend on context (i.e., the behavioral relevance of sensory information).

### Force and cortical modulations are not accompanied by startle-like responses

To test whether the stimuli elicited a startle response, in Experiments 2 and 3, we also recorded EMG activity from a number of muscles, both necessary and unnecessary for exerting force on the transducer. A startle response would activate some of the recorded muscles not necessary for the force exertion task, such as the sternocleidomastoid, whose activation is a core component of the startle reflex (Brown et al., 1991).

**Figure 7.**
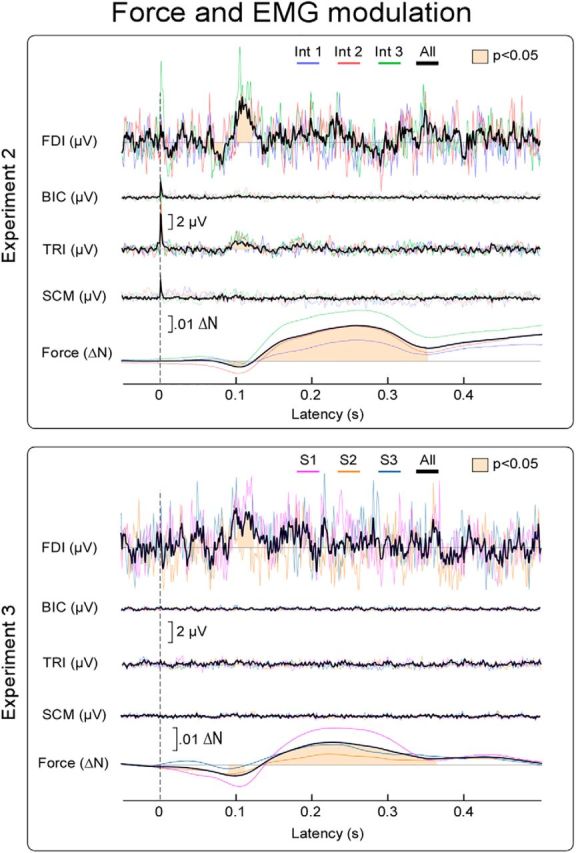
Force and EMG modulations. Group-average force magnitude and EMG activity recorded from the right FDI, biceps (BIC), triceps (TRI), and sternocleidomastoid (SCM) muscles, following somatosensory stimulation of the left median nerve (Experiment 2, three intensities; top) or following auditory stimuli (Experiment 3, three consecutive stimuli; bottom). The displayed EMG signals were rectified and baseline corrected. The force decrease, peaking ∼100 ms after stimulus, was preceded by a reduction of EMG activity ∼75 ms after stimulus, only detectable in FDI. The force increase, peaking ∼250 ms after stimulus, was preceded by an increase of EMG activity ∼110 ms after stimulus, detectable in both FDI and TRI. These latencies are consistent with the electromechanical delay between EMG activity and changes in muscle tension (Eliasmith and Anderson, 2004; Graben et al., 2007).

In Experiment 2, the EMG activity of the first dorsal interosseous muscle (FDI; i.e., the muscle contributing to the force exerted on the transducer) showed two small but significant amplitude modulations: a reduction of amplitude at 70–80 ms after stimulus, followed by an increase of amplitude at 100–120 ms after stimulus (Fig. 7, first trace). The EMG amplitude increase was also observed in the triceps (95–120 ms after stimulus; Fig. 7, third trace). These EMG effects were temporally related to the first two modulations of the exerted force (Fig. 7, fifth trace), given the electromechanical delay between EMG activity and changes in muscle tension (Eliasmith and Anderson, 2004; Graben et al., 2007). Importantly, we observed no EMG response in the sternocleidomastoid muscle (Fig. 7, fourth trace). The lack of sternocleidomastoid activation rules out that the applied somatosensory stimuli elicited a startle response.

In Experiment 3, the results were very similar to what was observed in Experiment 2: auditory stimuli did not elicit any clear response in the EMG recorded from the biceps and sternocleidomastoid muscles, whereas the EMG activity of the FDI and triceps muscles showed a significant increase of amplitude at 99–110 ms (triceps) and 100–120 ms (FDI) after stimulus. One-way ANOVA showed a weak suggestion that the increase in EMG activity recorded from the FDI was higher in S1 than in S2 and S3 (main effect of “stimulus repetition”: *F*_(2,26)_ = 2.77, *p* = 0.088). The same analysis applied to the EMG recorded from the triceps did not show evidence for any effect of “stimulus repetition” (*F*_(2,26)_ = 0.43, *p* = 0.65), possibly because of the much smaller signal-to-noise ratio of the stimulus-evoked modulation of EMG compared with force.

Together, these results indicate that the applied somatosensory or auditory stimuli did not elicit a startle response. However, to test more explicitely the dissociation between the observed force modulation and a startle-like response, in Experiment 4 we compared the force and EMG modulations elicited by standard and startling stimuli.

**Figure 8.**
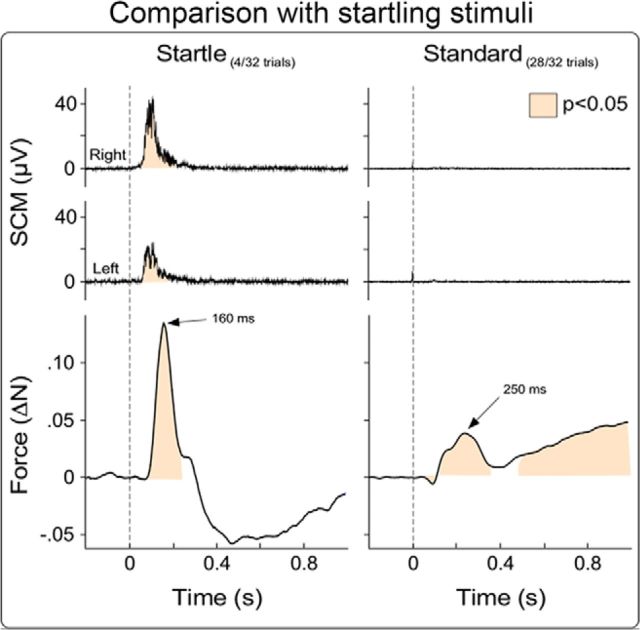
Force and EMG modulations following startling and standard stimuli. Group-average force magnitude (bottom) and EMG activity (top) recorded from left and right sternocleidomastoid (SCM) muscles, following startling auditory stimuli (4 trials, left) and standard somatosensory stimuli (28 trials, right). EMG signals were rectified and baseline corrected. Unlike standard stimuli, the startling stimuli evoked a clear startle reflex, reflected in the EMG response observed in both the left and the right SCM. Furthermore, the force modulation pattern following startling stimuli consisted of a unipolar force increase peaking ∼160 ms after stimulus.

The results from Experiment 4 provided clear evidence that nonstartling stimuli are sufficient to elicit the force modulation observed in Experiments 1–3. Indeed, while the same standard stimuli used in Experiments 1–3 did not elicit EMG responses in the sternocleidomastoid muscle, such EMG responses were clearly elicited by startling stimuli (Fig. 8). Furthermore, startling stimuli elicited a consistent unipolar force increase (133 ± 116 mN), peaking ∼160 ms following stimulus onset (Fig. 8) (i.e., a pattern different from the multipolar force modulation elicited by standard stimuli; Fig. 2).

## Discussion

Here we describe a basic physiological phenomenon that couples saliency-related cortical responses to motor output. Salient sensory stimuli modulate ongoing force exerted by human subjects, and this modulation is tightly linked to the electrocortical activity elicited by the same stimuli. We obtained four main results. First, the force modulation follows a complex triphasic pattern consisting of alternating decreases and increases of force, time-locked to stimulus onset. Second, this modulation occurs regardless of the sensory modality of the eliciting stimulus. Third, the magnitude of the force modulation is predicted by the amplitude of the cortical activity elicited by the same stimuli. Fourth, the stimulus-evoked force modulations, as well as the cortical responses, are not stereotyped, but their magnitudes strongly depend on stimulus saliency. Together, these results indicate that sudden environmental changes have a clear effect on motor reactivity, possibly to prepare subsequent actions. This phenomenon is subserved by a tight coupling between stimulus-evoked cortical responses and motor output.

### Force modulation follows a complex pattern

In all four experiments, we observed that sudden sensory stimuli evoked force modulations in a complex pattern in nearly every participant tested (Fig. 3). The complexity of this pattern is incompatible with the unidirectional, nonsequential, atomic nature of reflex responses. In particular, it does not fit the criteria of a startle reflex, which is defined as a generalized flexion response with a sustained increase of EMG activity in facial, neck, and shoulder muscles (e.g., Fig. 8), only elicited by unexpected stimuli delivered at extremely long intervals (e.g., in the order of minutes) (Wilkins et al., 1986; Brown et al., 1991; Aramideh and Ongerboer de Visser, 2002). In contrast, we observed the following: (1) alternating patterns of decreases and increases of muscular activity; (2) absence of EMG responses in the sternocleidomastoid muscle (which is one of the most robust components of the startle; Figs. 7, 8) (Brown et al., 1991); (3) EMG response in extensor (triceps) rather than flexor (biceps) muscles; and (4) presence of a response even at interstimulus intervals as short as 5–10 s (Experiments 1 and 2) and 1 s (Experiment 3).

The force pattern we observed is also different from the unipolar increases of exerted force during voluntary goal-directed isometric contractions (Desmedt and Godaux, 1978; Ferrari-Toniolo et al., 2015). Instead, the alternating patterns of force decreases and increases are reminiscent of the earliest stage of voluntary ballistic movements, when agonists and antagonists muscles alternate bursts of activity to trigger a goal-directed action (Marsden et al., 1983; Berardelli et al., 1996). These observations raise the intriguing possibility that the complex patterns in muscle activity lead to nonstereotyped behavior in response to sudden changes in the sensory environment (Graziano, 2008), an explanation that also justifies the energy consumption necessary for producing a muscular modulation lasting a few seconds.

### Force modulation is mediated by a supramodal mechanism

Our results suggest a supramodal modulation of motor neuron activity. Across the four experiments, we consistently observed that both somatosensory and auditory stimuli yield virtually identical modulations of force magnitude (Figs. 2, 3). In particular, the striking similarity of the response elicited by somatosensory and auditory stimuli indicates that the observed force modulation is not orchestrated by a spinal circuit but by supraspinal modulation of α motoneurons in the ventral horn.

This interpretation is further supported by the EMG recordings, in which modulations elicited by somatosensory and auditory stimuli were virtually identical. The clearest EMG modulation was in the FDI muscle, which is most directly involved in exerting force on the transducer (Fig. 7). However, these EMG modulations were not as clear as the force modulations, possibly because of the higher sensitivity of the force transducer compared with the EMG. While these EMG modulations could have been overlooked in previous investigations, few sparse observations in the literature are consistent with the supramodal nature of our observations. Transient decreases of EMG activity, at a comparable latency to our observations in FDI activity reduction, have been reported following both somatosensory and auditory stimulation (Rossignol and Jones, 1976; Delwaide and Schepens, 1995; Zehr et al., 2001; Kagamihara et al., 2003). However, these studies did not compare stimuli of different sensory modalities, and the observed modulations were interpreted in a modality-specific manner. Somatosensory modulations were interpreted as either propriospinal (Zehr et al., 2001) or long-loop brainstem (Kagamihara et al., 2003) reflexes, whereas loud auditory stimuli have been suggested to modulate EMG activity through a so-called “audio-spinal” pathway (Rossignol and Jones, 1976; Delwaide and Schepens, 1995). Instead, our observation of virtually identical force and EMG modulations in response to both somatosensory and auditory stimuli suggests a supramodal corticospinal mechanism prompting appropriate behavior in response to any salient environmental event (Figs. 2, 3, 4, 6).

### Cortical activity predicts stimulus-evoked force modulation

In two experiments, we observed that salient sensory stimuli evoked not only complex force modulations, but also large-amplitude transient cortical responses. These responses were dominated by typical negative-positive vertex waves (Bancaud et al., 1953), whose latencies were similar to those of the first decreases and increases of exerted force (Fig. 4). This observation suggests a tight relationship between the cortical and muscular activity. To further explore the relationship between cortical and muscular activity we correlated, in each subject, trial-by-trial EEG and force magnitude across the entire time course. This analysis showed that variability in cortical activity predicted later variability of force magnitude, up to almost 2 s (Fig. 5). This result suggests that cortical activity is not merely concomitant to but drives muscle contraction.

The spatial distribution of this correlation suggests an even richer interpretation. Considering that the topography of the vertex wave is, by definition, maximal and symmetrical around the scalp vertex (Fig. 4) (Mouraux and Iannetti, 2009), it was remarkable that the correlations between cortical activity and force modulation had a nonsymmetrical topography, clearly different from that of the vertex wave (Figs. 4, 5). These diverging correlation topographies indicate that the vertex wave might contribute to force modulation through physiological effects distinct in time and space. During the vertex negativity (110–180 ms), we observed a modulation maximal on the electrodes over the sensorimotor cortex contralateral to the somatosensory stimulus (Fig. 5*A*,*B*), suggesting an effect on the processing of the afferent sensory input. In contrast, during the vertex positivity (200–370 ms), we observed a modulation over the sensorimotor cortex ipsilateral to the somatosensory stimulus but contralateral to the hand exerting the force (Fig. 5*C*,*D*), suggesting a later effect on the efferent corticospinal drive. The topography of this second correlation, although rather spread, shows a maximum over a number of frontal electrodes (Fig. 5*C*,*D*). Given the existence of a number of premotor areas projecting directly to spinal motoneurons in addition to the primary motor cortex (Dum and Strick, 1991, 2002), it is tempting to speculate that these nonprimary corticospinal projections might be modulated by the vertex wave on the basis of the observed correlation topographies.

This discrepancy in the correlation topographies is richly informative about the physiological nature of this corticomuscular relationship, as it implies that the entire response (i.e., the vertex wave) does not correlate with the force modulation. Instead, either: (1) a specific subset of neural generators, active throughout the vertex wave, predict force modulation; (2) the vertex wave has an effect on the activity of specific cortical modules, which in turn modulate the exerted force; or (3) a subcortical structure drives both the vertex wave and the force modulations. We favor one of the first two explanations, as it is unlikely that a subcortical structure driving both the cortical and muscular responses could account for a trial-by-trial relationship that changes in direction (sign) and topography across the two peaks of the EEG potential (Fig. 5). This consideration also rules out that the observed force modulation is simply consequent to a distraction from the isometric motor task: had these modulations just been due to distraction, one would expect the relationship between EEG and force to be affected in a similar way, both with respect to direction and scalp topography. Direct coupling between cortical and muscular activity seems more parsimonious. This interpretation is also supported by the direct functional connections from the somatosensory to motor cortices following somatosensory stimulation in rodents and humans (Ferezou et al., 2007; van Ede et al., 2015; Avanzini et al., 2016).

### Force modulation is not stereotyped but depends on context

Our results imply that the nervous system modulates force depending on the context: specifically, when the stimulus is salient. In Experiment 3, we dissociated stimulus saliency from afferent sensory input (Iannetti et al., 2008; Zhang et al., 2012). Both low- and high-saliency stimuli elicited force modulation, but the modulation elicited by the former was of clearly reduced magnitude (Fig. 6, bottom). As expected, simultaneous EEG recordings showed that cortical activity was similarly dependent on contextual modulation by stimulus saliency (Fig. 6, top). Context-dependent force modulations, in contrast to reflexive or startle responses, likely reflect a neural system for purposeful behavior in response to unexpected environmental events (Sherrington, 1906).

This observation is particularly important to link the present results to other research exploring the effect of unexpected events on the motor system (for relevant theoretical frameworks, see Parmentier, 2014; Horstmann, 2015; Wessel and Aron, 2017). In particular, “stopping” or “slowing” motor behaviors are commonly observed following action errors (Ruiz et al., 2009), unexpected action outcomes (Gentsch et al., 2009) or surprising perceptual events (Horstmann, 2006; Wessel and Aron, 2013). These behaviors are associated with a reduction of corticospinal excitability following the unexpected event. It is tempting to speculate that this reduction of corticospinal excitability (which occurs as early as 150 ms) (Wessel and Aron, 2013) and our early force decrease (Fig. 2) might share a common physiological mechanism.

### The vertex wave reflects a sensorimotor process

The human brain responds to sudden, intense, and behaviorally relevant stimuli with one of the largest synchronizations of electrocortical activity measurable from the scalp surface using EEG. This large biphasic vertex wave has been traditionally described as a correlate of perceptual processes (Carmon et al., 1978; Chapman et al., 1981), and later interpreted as reflecting the detection of salient stimuli (Iannetti et al., 2008; Mouraux and Iannetti, 2009). Preliminary evidence suggests that the vertex wave might be related to executing rapid defensive movements (Moayedi et al., 2015).

Our results indicate that the vertex wave is better conceptualized as a context-dependent sensorimotor process. Salient changes in the sensory environment, regardless of their modality, elicit cortical vertex waves directly affecting muscles, which resonate in similar patterns of force amplitude changes. Variations in vertex wave amplitude, either spontaneous or obtained through a dedicated experimental modulation, reliably predict force modulations. This suggests that such “corticomuscular resonance” is obligatory, as the stimulus-evoked force modulations cannot be dissociated from the cortical vertex waves. More specifically, the fact that the cortical and muscular responses appear to be both (1) obligatorily coupled and (2) coupled with a strength that further depends on the context, points toward a plausible evolutionary advantage of this response. As such, this phenomenon might represent a direct link between sensory and motor processes, with the objective of preparing muscles to respond appropriately to current or future sensory input, prompting a reinterpretation of saliency detection as a reactive rather than a perceptive process.
